# Changes in the health literacy of residents aged 15–69 years in central China: A three-round cross-sectional study

**DOI:** 10.3389/fpubh.2023.1092892

**Published:** 2023-02-24

**Authors:** Xin Mei, Gong Chen, Yuting Zuo, Qian Wu, Junlin Li, Yilin Li

**Affiliations:** ^1^Wuhan Center for Disease Control and Prevention, Wuhan, China; ^2^Medical Department, The Central Hospital of Wuhan, Tongji Medical College, Huazhong University of Science and Technology, Wuhan, China

**Keywords:** health literacy, changing trend, prevalence, healthcare, COVID-19

## Abstract

**Introduction:**

Health literacy is an effective strategy to promote more cost-effective use of health care services and a crucial tool for preventing the spread of infectious diseases. The main objective of this study was to analyze changes in health literacy from 2019 to 2021.

**Methods:**

Data were from the latest three-round cross-sectional studies with the same study design.

**Results:**

Although the prevalence of adequate health literacy rose significantly over time, increasing from 26.9% (95% CI 20.1–33.7) in 2019 to 34.1% (32.9–35.3) in 2021, it was still at a relatively low level. The most significant decrease was observed for health skills among the three aspects and health information literacy among the six dimensions. Working as medical staff was a protective factor for adequate health literacy, health skills literacy, and health information literacy. Risk factors for adequate health literacy and health information literacy were older age and lower education level. Furthermore, adequate health information literacy was positively related to annual family income.

**Discussion:**

More practical and effective policies targeting health literacy for critical aspects and groups in Central China, are urgently needed, especially during the epidemic.

## Introduction

As defined by the U.S. National Library of Medicine and the World Health Organization (WHO), health literacy refers to the ability to obtain, understand, and process basic health information and services and use them to make sound health-related decisions to maintain and promote health (1, 2). Health literacy is an essential factor affecting health and a strong predictor of the population's health status (3). Studies have shown that limited health literacy is not only related to adverse health behaviors such as smoking, alcoholism, low physical activity, difficulty communicating with doctors, and poor adherence to medicines prescribed by doctors but also closely related to adverse health outcomes such as hypertension, diabetes, stroke, and high mortality (4–8). Limited health literacy will also lead to increased medical expenses and waste of healthcare services (9). Thus, improving health literacy can be an effective strategy to promote more cost-effective use of healthcare services, contributing to the ultimate goal of primary healthcare and improving the population's health (10–14).

Therefore, the international emphasis on health literacy is increasing (15–17). Health literacy has become a research hotspot in clinical medicine, health education, and health promotion (18, 19). Research on health literacy is mainly based on two perspectives: the clinical perspective (20) and the public health perspective (21).

Research on health literacy in North American and European countries mainly focuses on the clinical perspective (20, 21). With the development of the health literacy evaluation system, many countries have successively launched health literacy surveys. The setting for these studies has been expanded from patients to the general public, and the measurement content has expanded from the clinical context to disease prevention, healthcare, and health promotion (22).

From the perspective of public health, the impact of health literacy on disease prevention, healthy lifestyle and behavior, and maintenance and promotion of health was studied in China (23). The National Health Commission of China released the educational book “Health Literacy of Chinese Citizens-Basic Knowledge and Skills (Trial)” and organized the first national health literacy survey in 2008 (24). In 2010, the China Health Education Center studied the evaluation system of health literacy, with the educational book as the evaluation content, and compiled the Chinese Health Literacy Survey Questionnaire (CHLSQ) (25). Since 2012, China has carried out scientific and continuous health literacy monitoring. Focusing on basic knowledge and concepts, healthy lifestyle and behavior, and health skills, a health literacy monitoring system for permanent residents aged 15–69 has gradually been established in China (23).

Although the definitions, measurement instruments, evaluation perspectives, and survey methods of health literacy are different in different countries or regions, many surveys have come to the same conclusion: Globally, health literacy needs to be improved (26, 27). The National Assessment of Adult Literacy (NAAL) found that 88% of adults do not have sufficient health literacy in the USA (26). A systematic review indicated that the prevalence of low health literacy ranged from 27 to 48% in Europe (27). The Chinese national health literacy survey showed that the prevalence of adequate health literacy among residents aged 15–69 was only 19% (28).

On 31 January 2020, the WHO declared the COVID-19 outbreak a Public Health Emergency of International Concern (17). The pandemic posed a considerable threat to human health (29, 30). Health literacy is a crucial determinant of health at both the social and individual levels, in healthy populations and with diverse infectious diseases (31), which is also a crucial tool for preventing the spread of infectious diseases (32).

Previous studies have highlighted the significance of health literacy for the outcomes of infectious diseases and the role that health literacy plays regarding infectious diseases (33, 34). People with low health literacy may not obtain adequate health knowledge on time and cannot implement protective behaviors, such as the adoption of immunization, to prevent infectious diseases (33). Therefore, it is significant to study the changes in the health literacy level and its determinants during this time. However, we found few studies describing the changes in health literacy during the pandemic.

Wuhan is located in central China, with a permanent population of 13.6 million (35). The main objective of this study was to analyze, based on three-wave city-level representative data among 15- to 69-year-old permanent residents in Wuhan, China, levels and changes in health literacy from 2019 to 2021 and the relationship between health literacy and related factors.

## Materials and methods

### Study population

The China Health Literacy Survey (CHLS) is a nationally representative household survey of the permanent population aged 15–69 (36). In conjunction with the CHLS, the Wuhan Health Literacy Survey (WHLS) aimed to provide data necessary to estimate health literacy since 2016 at the 1-year interval. The WHLS is a cross-sectional survey using the CHLS standardized protocol and questionnaire. We based our study on the latest three rounds (conducted from August to November 2019, 2020, and 2021) of the WHLS. The processes and sampling design of the survey were reviewed and approved by the Institutional Review Board (IRB) of Wuhan CDC (WHCDCIRB-K-2019016). All study participants provided electronic informed consent. All collected data were anonymous and self-administered.

### Sampling method

The sample size was calculated by the formula $N=\frac{\mu_{\alpha }^{2}\times p\left(1-p\right)}{\delta^{2}}\times deff$, where α was the significance level, μ_α_ was the α-quantile of the standard normal distribution, *p* was the health literacy level, δ was the maximum permissible error, and *deff* was the design effect of complex sampling. Considering the rate of invalid questionnaires and rejections, the final sample size is expected to be calculated. The sampling procedure involved five stages to ensure the representativeness of the selected study population. First, the simple random sampling (SRS) method was used to select several administrative districts (six in 2019 and 2020 and five in 2021) from the 15 districts in Wuhan. Second, the SRS method was used in each administrative district to select several streets (four in 2019 and 2021 three in 2020). Third, the SRS method was used in each street to select several neighborhood committees (three in 2019 and 2021 and two in 2020). Fourth, the SRS method was used in each neighborhood committee to select several households (55 in 2019, 85 in 2020, and 80 in 2021). Fifth, one resident was selected from each household using the KISH method, and a certain number of questionnaires were completed in each neighborhood committee (40 in 2019, 70 in 2020, and 52 in 2021).

### Measurement instrument

The CHLSQ, as compiled by the China Health Education Center (36), was used to measure health literacy. The questionnaire has strong internal consistency and split-half reliability (23), which consists of two parts: sociodemographic characteristics and health literacy content (a total of 50 items). The 50 items include eight true-or-false questions, 23 single-choice questions, 15 multiple-choice questions, and four situational questions (including three single and one multiple-choice questions). The 50-item health literacy is further categorized into three aspects and six dimensions. Based on the knowledge, attitude, practice (KAP) theory, the three aspects of literacy are basic knowledge and concept literacy, healthy lifestyles and behavior literacy, and health skill literacy (25). Guided by public health problems, the six dimensions of literacy are scientific views of health, infectious disease literacy, chronic disease literacy, safety and first aid literacy, medical care literacy, and health information literacy (24).

The total score of 50 items ranged from 0 to 66 points, with one point for every true-or-false and every single-choice question and two points for every multiple-choice question. Moreover, every wrong or missing choice received 0 points. The total scores of the three aspects were 28 (basic knowledge and concepts literacy, 22 items), 22 (healthy lifestyles and behavior literacy, 16 items), and 16 (health skill literacy, 12 items) points. The maximum total scores for the six dimensions of literacy were 11 points (scientific views of health, eight items), seven points (infectious disease literacy, six items), 12 points (chronic disease literacy, nine items), 14 points (safety and first aid literacy, ten items), 14 points (medical care literacy, 11 items), and eight points (health information literacy, six items).

Adequate health literacy is defined as when participants achieve more than 80% of the total score (53–66 points), and limited health literacy is defined as when participants score <80% of the total score (0–52 points) (24, 25). The judgment criterion for adequate health literacy in each aspect or dimension was ≥80% of the total score for the aspect or dimension. Health literacy level was defined as the proportion of participants who had adequate health literacy out of the total number of participants, as was the health literacy level of the three aspects and six dimensions (37).

### Survey method

Before the fieldwork, the neighborhood committee issued an investigation announcement about the purpose of the study to encourage residents to participate. In the investigation, face-to-face interviews were conducted at each participant's home or in other public places at their convenience. A portable tablet was used to complete electronic questionnaires. If participants could not complete the questionnaire, the investigators would neutrally interview them as an alternative to completing the questionnaire on behalf of the participants. In addition, participants were sent small gifts as an incentive for participating. If the individuals were already participants, they could withdraw at any time without penalty or loss of benefits. Strict quality control was applied to the whole investigative process. Two training sessions were held, and all staff participated and passed the on-site exams. The investigator complied with the investigation guidelines during all processes.

### Statistical analysis

We used the following independent variables drawn from the literature in our analysis: (21, 24, 25, 38) sociodemographic characteristics (i.e., gender, age, marital status, education level, occupation, and annual family income) and self-reported health status (Supplementary Table 1).

Data cleansing rules were created to ensure accuracy and eliminate internal inconsistencies. The sampling weight was considered since the survey adopted a multi-stage sampling procedure. The three waves of data were weighted: calculation of the sampling weight, non-response adjustment, and poststratification calibration adjustment of the sample totals to the known population totals. All of the analyses were based on a complex survey design. Rao-Scott chi-square tests were used to compare the differences in health literacy among subgroups in bivariate analyses. Cochran–Armitage trend tests were used to measure trends in health literacy over time. Multivariable logistic regression analysis was conducted to identify the risk factors related to adequate health literacy. A two-sided 5% significance level assessed statistical inferences. Data cleaning, weighting, and analysis were conducted using SAS software version 9.4 (SAS Institute Inc. Cary, NC).

## Results

### Participant characteristics

Table 1 shows descriptive statistics of the study population over time. A total of 2,880 individuals in 2019, 2,520 individuals in 2020, and 3,120 individuals in 2021 were invited to participate in the survey, with effective response rates of 94.7% in 2019 (2,544 individuals), 95.3% in 2020 (2,295 individuals), and 99.0% in 2021 (3,088 individuals).

**Table 1:** Descriptive statistics of the study population over time (*n* = 7,927).

**Characteristic**	**Survey year 2019 (*****n*** = **2,544)**		**Survey year 2020 (*****n*** = **2,295)**		**Survey year 2021 (*****n*** = **3,088)**		**P^*c*^**
	*n* ^a^	**% (95%CI)^b^**	*n* ^a^	**% (95%CI)^b^**	*n* ^a^	**% (95%CI)^b^**	
**Gender**							
Male	1,238	51.3 (45.5–57.1)	1,110	51.3 (44.3–58.3)	1,492	51.3 (47.2–55.4)	
Female	1,306	48.7 (42.9–54.5)	1,185	48.7 (41.7–55.7)	1,596	48.7 (44.6–52.8)	
**Age, years**							
15–29	281	38.0 (32.7–43.4)	202	38.0 (28.8–47.2)	352	38.0 (30.6–45.4)	
30–44	830	28.2 (21.9–34.5)	629	28.2 (21.6–34.8)	889	28.2 (24.8–31.6)	
45–59	841	25.1 (19.2–31.1)	794	25.1 (16.8–33.4)	1,082	25.1 (20.8–29.5)	
60–69	592	8.7 (5.4–11.9)	670	8.7 (4.5–12.8)	765	8.7 (4.8–12.6)	
**Marital status**							<0.001
Unmarried	249	26.2 (18.2–34.3)	203	24.3 (14.5–34.0)	401	30.1 (22.8–37.5)	
Married	2,160	70.8 (61.7–80.0)	1,953	73.3 (63.6–83.0)	2,479	66.4 (59.4–73.5)	
Divorced/Widowed	135	2.9 (1.0–4.9)	139	2.5 (1.0–3.9)	208	3.4 (2.8–4.1)	
**Education level**							<0.001
College or above	790	43.5 (22.2–64.9)	688	48.0 (29.5–66.5)	1,011	46.8 (24.7–68.8)	
Senior high school and below	1,754	56.5 (35.1–77.8)	1,607	52.0 (33.5–70.5)	2,077	53.2 (31.2–75.3)	
**Occupation**							<0.001
Medical staff	47	2.3 (0.8–3.8)	53	3.0 (0.4–5.5)	69	2.7 (0.8–4.6)	
Civil servant/teacher	136	4.7 (1.4–7.9)	62	2.9 (1.1–4.7)	61	2.2 (1.2–3.1)	
Farmer/worker	788	18.5 (4.9–32.0)	829	23.4 (0.0–47.4)	943	24.1 (0.0–50.5)	
Others	1,573	74.6 (63.5–85.6)	1,351	70.7 (46.4–95.0)	2,015	71.0 (46.3–95.8)	
**Annual family income (CNY)** ^d^							<0.001
≥100,000	1,084	48.6 (25.4–71.8)	779	49.5 (26.2–72.8)	1,184	45.0 (24.8–65.2)	
< 100,000	1,460	51.4 (28.2–74.6)	1,516	50.5 (27.2–73.8)	1,904	55.0 (34.8–75.2)	
**Self-reported health status**							<0.001
Good	1,816	76.5 (67.6–85.3)	1,798	86.0 (81.0–91.0)	2,478	86.2 (81.8–90.7)	
Medium	658	21.4 (13.7–29.1)	450	12.7 (8.3–17.2)	518	11.7 (8.6–14.8)	
Poor	70	2.2 (0.8–3.5)	47	1.3 (0.2–2.4)	92	2.0 (0.3–3.7)	
**Health literacy**							<0.001
Limited (40–52 points)	1,948	73.1 (66.3–79.9)	1,648	66.4 (59.5–73.3)	2,175	65.9 (64.7–67.1)	
Adequate (53–66 points)	596	26.9 (20.1–33.7)	647	33.6 (26.7–40.5)	913	34.1 (32.9–35.3)	

a Unweight frequency.

b Weighted percentage. 95% CI, 95% confidence interval.

c On the basis of chi-square tests.

d CNY, Chinese Yuan; 1 US dollars, 6.7 Chinese yuan.

The unweighted average ages in 2019, 2020, and 2021 were 46.9 ± 13.4, 49.5 ± 13.7, and 47.8 ± 13.9, respectively. The male:female ratios in 2019, 2020, and 2021 were 0.95:1, 0.94:1, and 0.93:1, respectively. No statistically significant difference was found in the gender or age composition of the participants among the different years.

### Bivariate analysis of health literacy level with variables of sociodemographic characteristics

As shown in Table 2, there were significant differences in health literacy level by age, education level, and occupation but not by gender or self-reported health status in 2019, 2020, and 2021.

**Table 2:** Factors related to adequate health literacy (AHL) over time—results of bivariate analyses and trend analysis (weighted).

**Characteristic**	**AHL (Survey year 2019)**		**AHL (Survey year2020)**		**AHL (Survey year 2021)**		***Z***	***P*^b^for Trend**
	**%(95%CI)**	***P** ^a^*	**%(95%CI)**	***P** ^a^*	**%(95%CI)**	***P** ^a^*		
Gender		0.914		0.225		0.725		
Male	26.8 (19.3–34.3)		35.3 (26.0–44.6)		33.6 (28.7–38.5)		212.172	<0.001
Female	27.0 (20.1–34.0)		31.8 (25.7–37.8)		34.7 (30.6–38.7)		235.174	<0.001
Age, years		<0.001		<0.001		<0.001		
15–29	31.8 (18.5–45.0)		36.7 (26.4–47.0)		38.4 (34.0–42.7)		172.675	<0.001
30–44	29.6 (25.1–34.1)		41.5 (30.5–52.6)		36.9 (30.2–43.5)		164.221	<0.001
45–59	21.3 (15.7–27.0)		23.7 (16.6–30.9)		30.8 (23.1–38.4)		221.557	<0.001
60–69	13.2 (7.8–18.6)		22.7 (13.3–32.2)		16.4 (11.9–21.0)		51.455	<0.001
Marital status		0.625		0.022		0.041		
Unmarried	28.5 (16.4–40.7)		32.0 (22.0–42.0)		38.7 (32.2–45.2)		233.494	<0.001
Married	26.5 (21.4–31.6)		34.6 (28.3–40.9)		32.5 (28.1–37.0)		223.990	<0.001
Divorced/Widowed	23.2 (7.6–38.8)		18.8 (6.1–31.5)		25.6 (20.3–30.9)		22.414	<0.001
Education level		<0.001		0.042		0.001		
College or above	37.2 (24.4–50.0)		40.3 (35.8–44.8)		44.5 (38.6–50.5)		204.254	<0.001
Senior high school and below	19.0 (12.3–25.7)		27.4 (14.0–40.8)		25.0 (15.8–34.2)		216.982	<0.001
Occupation		<0.001		<0.001		<0.001		
Medical staff	63.0 (34.7–91.2)		63.5 (54.5–72.4)		72.0 (46.8–97.2)		63.337	<0.001
Civil servant/teacher	39.4 (20.2–58.5)		60.2 (27.0–93.4)		40.4 (11.4–69.4)		42.213	<0.001
Farmer/worker	19.1 (12.8–25.4)		22.9 (13.3–32.5)		29.1 (23.7–34.5)		220.361	<0.001
Others	26.9 (21.0–32.9)		34.8 (26.6–43.0)		34.2 (30.9–37.5)		272.719	<0.001
Annual family income (CNY)^c^		0.003		0.001		0.178		
≥100,000	32.7 (30.3–35.1)		42.2 (31.3–53.1)		36.8 (33.4–40.1)		123.490	<0.001
< 100,000	21.5 (10.8–32.1)		25.2 (17.0–33.3)		32.0 (25.9–38.0)		354.688	<0.001
Self-reported health status		0.699		0.636		0.067		
Good	27.6 (19.9–35.4)		33.3 (26.0–40.6)		35.3 (34.9–35.7)		297.991	<0.001
Medium	24.4 (17.2–31.6)		36.5 (21.8–51.2)		27.5 (17.5–37.6)		88.458	<0.001
Poor	27.0 (0.0–58.7)		24.8 (5.8–43.8)		22.6 (0.0–45.5)		−29.843	<0.001

AHL, adequate health literacy.

a Based on chi-square tests for comparing the proportion of adequate health literacy across groups.

b Based on Cochran–Armitage tests for comparing the changing trend of adequate health literacy across subgroups.

c CNY, Chinese Yuan; 1 US dollars, 6.7 Chinese yuan.

#### Trend analysis of health literacy, three aspects, and six dimensions of literacy over time

Table 2 shows the level and trend in health literacy for subgroups of sociodemographic characteristics. The prevalence of adequate health literacy in most subgroups showed a significant upward trend, but the subgroup of poor self-reported health status showed a significant downward trend from 2019 to 2021.

The level and trend of health literacy, the three aspects, and the six dimensions of literacy over time are presented in Table 3. The prevalence of adequate health literacy rose significantly over time, increasing from 26.9% (95% CI 20.1–33.7) in 2019 to 34.1% (32.9–35.3) in 2021.

**Table 3:** Adequate health literacy, three aspects, and six dimensions of literacy in total and subgroup population over time—results of trend analysis (weighted) (95% CI).

	**Survey year 2021**	**Survey year 2020**	**Survey year 2019**	**Z**	**P^a^ for Trend**
Health literacy	34.1 (32.9–35.3)	33.6 (26.7–40.5)	26.9 (20.1–33.7)	316.001	<0.001
**Three aspects**					
Basic knowledge and concepts	46.3 (42.1–50.5)	39.6 (27.2–52.0)	45.8 (32.4–59.1)	23.539	<0.001
Healthy lifestyles and behavior	41.1 (36.4–45.7)	38.7 (30.5–46.8)	27.8 (19.7–36.0)	561.653	<0.001
Health skills	26.5 (23.4–29.6)	29.6 (21.5–37.7)	31.1 (20.1–42.1)	−207.328	<0.001
**Six dimensions**					
Scientific views of health	57.3 (48.5–66.1)	55.9 (44.0–67.9)	59.7 (49.8–69.6)	−97.339	<0.001
Infectious disease literacy	39.4 (28.1–50.7)	39.9 (31.6–48.2)	19.8 (12.9–26.7)	848.035	<0.001
Chronic disease literacy	37.5 (32.5–42.5)	32.7 (25.1–40.2)	37.2 (26.8–47.5)	13.836	<0.001
Safety and first aid literacy	61.4 (50.2–72.7)	61.3 (50.2–72.4)	64.6 (53.1–76.2)	−134.418	<0.001
Medical care literacy	34.2 (26.3–42.1)	34.4 (24.6–44.3)	27.8 (17.1–38.5)	277.968	<0.001
Health information literacy	39.8 (29.5–50.2)	44.2 (35.5–53.0)	45.9 (30.5–61.3)	−249.012	<0.001

a Based on Cochran–Armitage tests, comparing changing trend of adequate health literacy in the total population and age subgroups.

In 2021, the lowest prevalence of adequate health literacy of the three aspects was for health skills; the lowest prevalence of the six dimensions was for medical care literacy.

In the trend analysis, the most significant increase was observed for healthy lifestyles and behavior (increased 39% in 2020 and 48% in 2021) among the three aspects and infectious disease literacy (increased 101% in 2020 and 99% in 2021) among the six dimensions; the most significant decrease was observed for health skills (decreased 15% in 2021) among the three aspects and health information literacy (decreased 13% in 2021) among the six dimensions.

### Multivariable logistic regression analysis of health literacy, health skill literacy, and health information literacy

As the most significant decrease was observed for health skills among the three aspects and health information literacy of the six dimensions, they were also included in the multivariable logistic regression analysis along with health literacy (Table 4).

**Table 4:** Multivariable logistic regression analysis of factors related to health literacy, health skill literacy, and health information literacy (weighted).

**Variables**	**Health literacy**		**Health skills literacy**		**Health information literacy**	
	**OR (95% CI)** ^a^	***P***	**OR (95% CI)** ^a^	***P***	**OR (95% CI)** ^a^	***P***
**Year**						
2021	Ref		ref		ref	
2020	0.9 (0.7–1.3)	0.691	1.1 (0.8–1.7)	0.528	1.2 (0.7–1.9)	0.510
2019	0.7 (0.5–0.9)	0.005	1.3 (0.9–1.9)	0.242	1.3 (0.8–2.1)	0.285
**Gender**						
Male	Ref		ref		ref	
Female	0.9 (0.8–1.1)	0.245	0.9 (0.7–1.1)	0.309	1.0 (0.9–1.1)	0.930
**Age, years**						
15–29	Ref		ref		ref	
30–44	0.9 (0.7–1.2)	0.575	1.0 (0.8–1.3)	0.905	0.9 (0.7–1.0)	0.131
45–59	0.7 (0.5–1.0)	0.024	0.7 (0.5–1.0)	0.083	0.7 (0.6–0.8)	<0.001
60–69	0.5 (0.3–0.7)	<0.001	0.5 (0.3–0.8)	0.003	0.5 (0.4–0.7)	<0.001
**Marital status**						
Unmarried	Ref		ref		ref	
Married	1.2 (0.9–1.7)	0.158	1.1 (0.8–1.5)	0.724	1.2 (0.9–1.5)	0.146
Divorced/Widowed	1.1 (0.7–1.8)	0.704	0.8 (0.6–1.2)	0.344	1.1 (0.8–1.5)	0.546
**Education level**						
College or above	Ref		ref		ref	
Senior high school and below	0.6 (0.4–0.9)	0.011	0.7 (0.5–1.1)	0.099	0.6 (0.5–0.8)	<0.001
**Occupation**						
Medical staff	Ref		ref		ref	
Civil servant/teacher	0.4 (0.2–0.7)	0.001	0.4 (0.2–0.6)	<0.001	0.5 (0.3–0.7)	<0.001
Farmer/worker	0.3 (0.2–0.4)	<0.001	0.3 (0.2–0.4)	<0.001	0.4 (0.2–0.6)	<0.001
Others	0.3 (0.2–0.4)	<0.001	0.3 (0.2–0.4)	<0.001	0.4 (0.2–0.6)	<0.001
**Annual family income (CNY)** ^b^						
≥100,000	Ref		ref		ref	
< 100,000	0.8 (0.5–1.0)	0.078	0.8 (0.6–1.2)	0.308	0.8 (0.6–1.0)	0.021
**Self-reported health status**						
Good	Ref		ref		ref	
Medium	1.1 (0.9–1.4)	0.297	1.0 (0.8–1.2)	0.991	1.0 (0.8–1.1)	0.582
Poor	1.0 (0.6–1.7)	0.984	1.1 (0.7–1.7)	0.705	0.7 (0.4–1.3)	0.295

a OR, odds ratio; 95% CI, 95% confidence interval;

b CNY, Chinese Yuan; 1 US dollars, 6.7 Chinese yuan.

Compared to 2021, the odds of adequate health literacy were significantly lower in 2019. Working as medical staff was a protective factor for adequate health literacy, health skill literacy, and health information literacy compared with other occupations. Risk factors for adequate health literacy and health information literacy were older age (45–69) and lower education level (senior high school and below). Risk factors for adequate health skill literacy were older age (60–69). Furthermore, adequate health information literacy was positively related to annual family income.

## Discussion

This is the first study describing the changes over time in health literacy in Wuhan, central China, based on representative three-time-series survey data. We observed that the prevalence of adequate health literacy rose significantly over time, increasing from 26.9% (95% CI 20.1–33.7) in 2019 to 34.1% (32.9–35.3) in 2021. Although the prevalence showed the same upward trend as a previous study (37) and is slightly higher than that of the Chinese national level (25.4%) (39), it is still at a relatively low level, similar to American and European countries (26, 27). The significant rise may be mainly related to economic and social development, the in-depth development of health education and health promotion, and the people's close attention to and urgency regarding health during the COVID-19 epidemic (24, 37, 40, 41).

In 2021, the highest prevalence of adequate health literacy among the three aspects was for basic knowledge and concepts, and the lowest was for health skills. The prevalence of adequate health literacy for healthy lifestyles and behaviors has risen rapidly, and health skills have shown a significant downward trend. In recent years, healthy lifestyle actions have been vigorously carried out, and knowledge of infectious diseases has been spread, effectively promoting healthy behavior (42). Health education should focus on behavioral intervention and health skill training in the future.

In 2021, the lowest prevalence among the six dimensions was medical care literacy. Residents who lack medical care literacy may not be able to access and understand basic health information and services and cannot effectively utilize the complex healthcare system when they seek treatment (2, 10). From the perspective of trend changes, the most significant increase was observed for infectious disease literacy among the six dimensions. It may be that the government and health departments paid more attention to educating the public about infectious disease prevention and control due to the COVID-19 epidemic (30). Against this background, people not only knew about virus transmission routes but also knew how to engage in effective preventive behaviors such as hand washing, mask-wearing, household ventilation and disinfection, and reduced interpersonal contact by avoiding visiting crowded spaces (42, 43). In addition, the prevalence of adequate literacy of the six dimensions for scientific views of health, safety and first aid, and health information showed a downward trend from 2019 to 2021, and health information literacy declined the most. Therefore, health education in Wuhan should focus on the aforementioned dimensions of literacy.

In multivariable logistic regression analysis, working as the medical staff was a protective factor for adequate health literacy, health skill literacy, and health information literacy compared with other occupations, which is in line with the characteristics of an occupation engaged in the medical and healthcare industries (23, 24, 44). The education level, knowledge reserve, and information acquisition channels of medical staff are better than those of other occupations. This study also showed that the prevalence of health literacy of residents who reported poor health status showed a significant downward trend from 2019 to 2021, indicating that medical staff can be used to carry out health education of residents with poor health status seeking treatment, to improve their health literacy in a targeted manner.

Risk factors for adequate health literacy and health information literacy were older age and lower education level, consistent with previous studies (24, 25, 37, 45). This may be due to the following reasons: the cognitive ability, learning ability, and memory of elderly people decline, and their ability to accept new knowledge is relatively poor, directly leading to the poor acquisition of health knowledge and skills and limited health literacy; well-educated individuals are more likely to seek beneficial information and medical care and can communicate effectively with healthcare workers (46). In addition, adequate health information literacy was positively related to annual family income, consistent with previous studies (21, 38, 47). This may be because a good economic situation positively affects the acquisition of health information and the utilization of healthcare resources. This indicates that targeted health education and health promotion should be strengthened, focusing on residents with older ages, lower education levels, and lower annual family incomes.

Our study has several limitations that can be improved in further research. First, the study design was cross-sectional, and no causal relationships could be made. Second, some factors, such as health behaviors, and health service quality were not assessed. Third, we obtained data from self-reported items, which are prone to bias. Finally, our research population consisted of permanent residents aged 15–69, and some groups were not included, which should be further studied.

This is the first study to characterize the levels, changes, and factors related to health literacy among residents aged 15–69 from 2019 to 2021 in central China. Overall, although the prevalence of adequate health literacy rose significantly, increasing from 26.9% (95% CI 20.1–33.7) in 2019 to 34.1% (32.9–35.3) in 2021, it was still at a relatively low level. In the context of the COVID-19 epidemic, the prevalence of adequate infectious disease literacy rose rapidly, but health skills and health information literacy declined. The protective factor for adequate health literacy, health skill literacy, and health information literacy was working as medical staff, and the risk factors were older age, lower education level, and lower annual family income. Tailored health education and promotion strategies are needed for different subgroups of residents to improve health literacy, especially for health skills and health information literacy. At the same time, medical staff with adequate health literacy can effectively be used by providing health education for people who seek treatment with a poor health status to improve the health literacy of this population.

## Data availability statement

The data analyzed in this study is subject to the following licenses/restrictions: The datasets generated during and/or analyzed during the current study are not publicly available due to restrictions applied to the availability of these data but are available from the corresponding author on reasonable request. Requests to access these datasets should be directed to liyilin@whcdc.org.

## Ethics statement

The processes and sampling design of the survey were reviewed and approved by the Institutional Review Board (IRB) of Wuhan CDC (WHCDCIRB-K-2019016). Written informed consent to participate in this study was provided by the participants' legal guardian/next of kin.

## Author contributions

XM: conceptualization, formal analysis, investigation, data curation, and writing—reviewing and editing. GC, YZ, and QW: formal analysis, investigation, and writing—reviewing and editing. JL: conceptualization, methodology, writing—reviewing and editing, supervision, and project administration. YL: conceptualization, methodology, investigation, writing—reviewing and editing, supervision, and project administration. All authors contributed to the article and approved the submitted version.
