# Supplementary material for: Changes in the health literacy of residents aged 15–69 years in central China: A three-round cross-sectional study
Source: Front Public Health. 2023 Feb 24;11:1092892. doi: 10.3389/fpubh.2023.1092892 (PMC9998942; doi:10.3389/fpubh.2023.1092892)
Supplement: Supplementary file 1 [file Table_1.DOCX]

Table Question-wording, response options and coding

| **Variables** | **Survey items** | **Original response options** | **Categorical variable measure** |
| --- | --- | --- | --- |
| **Dependent variable** |  |  |  |
| Adequate health literacy | 50 items, with a total score of 66 | Below basic (0-26) | Adequate (53-66)= yes= 1 |
|  |  | Basic (27-39) | Any other option (0-52)= no= 0 |
|  |  | Intermediate (40-52) |  |
|  |  | Adequate (53-66) |  |
| **Independent variables** |  |  |  |
| Gender | What's your gender? | Male | Male=0 |
|  |  | Female | Female=1 |
| Age, years | Date of birth: _____year ___month. | 15-69 | 15-29=1 |
|  |  |  | 30-44=2 |
|  |  |  | 45-59=3 |
|  |  |  | 60-69=4 |
| Marital status | What's your marital status? | Unmarried | Unmarried=1 |
|  |  | Married | Married/Separated =2 |
|  |  | Separated | Divorced/Widowed=3 |
|  |  | Divorced |  |
|  |  | Widowed |  |
| Educational level | What is your education level? | Illiterate | College/ Master's degree or above=College or above=0 |
|  |  | Primary school | Any other response=Senior high school and below= 1 |
|  |  | Junior high school |  |
|  |  | senior high school |  |
|  |  | College |  |
|  |  | Master's degree or above |  |
| Occupation | What is your occupation? | Civil servant | Medical staff= 1 |
|  |  | Teacher | Civil servant/ Teacher= 2 |
|  |  | Medical Staff | Farmer/ worker= 3 |
|  |  | Staff at other public institutions | Any other response= Others= 4 |
|  |  | Student |  |
|  |  | Farmer |  |
|  |  | Worker |  |
|  |  | Staff of other enterprises |  |
|  |  | Other |  |
| Annual family income (CNY)a | What was your family's annual income in the past year (Chinese Yuan, CNY)? | <30,000 | ≥100,000= 0 |
|  |  | 30,000-50,000 | <100,000= 1 |
|  |  | 50,000-100,000 |  |
|  |  | 100,000-300,000 |  |
|  |  | ≥300,000 |  |
| Self-reported health status | How would you rate your overall health during the past year? | Very good | Good/very good=Good = 1 |
|  |  | Good | Medium= 2 |
|  |  | Medium | Bad/very bad=Bad = 3 |
|  |  | Bad |  |
|  |  | Very bad |  |

* CNY=Chinese Yuan, 1 US dollars=6.7 Chinese yuan.
